# Chitosan-Coated Silver Nanocomposites: Biosynthesis, Mechanical Properties, and Ag^+^ Release in Liquid and Biofilm Forms

**DOI:** 10.3390/ijms26094130

**Published:** 2025-04-26

**Authors:** Daniel Martínez-Cisterna, Lingyun Chen, Leonardo Bardehle, Edward Hermosilla, Gonzalo Tortella, Manuel Chacón-Fuentes, Olga Rubilar

**Affiliations:** 1 Doctorado en Ciencias de Recursos Naturales, Facultad de Ciencias Químicas y Recursos Naturales, Universidad de La Frontera, Av. Francisco Salazar 01145, Casilla 54-D, Temuco 4811230, Chile; d.martinez11@ufromail.cl; 2 Centro de Investigación Biotecnológica Aplicada al Medio Ambiente (CIBAMA), Universidad de La Frontera, Av. Francisco Salazar 01145, Casilla 54-D, Temuco 4811230, Chile; leonardo.bardehle@ufrontera.cl (L.B.); edward.hermosilla@ufrontera.cl (E.H.); gonzalo.tortella@ufrontera.cl (G.T.); 3 Laboratorio de Química Ecológica, Departamento de Ciencias Químicas y Recursos Naturales, Universidad de La Frontera, Av. Francisco Salazar 01145, Casilla 54-D, Temuco 4811230, Chile; 4 Department of Chemical Engineering, Universidad de La Frontera, Av. Francisco Salazar 01145, Temuco 4811230, Chile; 5 Department of Agriculture, Food and Nutritional Sciences, University of Alberta, Edmonton, AB T6G 2P5, Canada; 6 Departamento de Producción Agropecuaria, Facultad de Ciencias Agropecuarias y Medioambiente, Universidad de La Frontera, Av. Francisco Salazar 01145, Casilla 54-D, Temuco 4811230, Chile; 7 Agriaquaculture Nutritional Genomic Center, CGNA, Temuco 4780000, Chile; manuel.chacon@cgna.cl

**Keywords:** biosynthesized nanoparticles, chitosan nanocomposite, silver nanoparticles

## Abstract

This study explores the biosynthesis, characterization, and evaluation of silver nanoparticles coated with chitosan (AgChNPs) for liquid nanocomposite and biofilm formation in integrated pest management (IPM). AgChNPs were synthesized using *Galega officinalis* leaf extract as a reducing agent, with varying chitosan concentrations (0.5%, 1%, and 2%) and pH levels (3, 4, and 5). Synthesis was optimized based on nanoparticle size, stability, and polydispersity index (PDI) over 21 days. Biofilms incorporating AgChNPs were analyzed for chemical, physical, mechanical, and thermal properties via Ultraviolet-visible spectroscopy (UV-vis), Dynamic Light Scattering (DLS), Zeta Potential Analysis, Fourier Transform Infrared Spectroscopy (FTIR), X-Ray Diffraction (XRD), Transmission Electron Microscopy with Energy Dispersive X-ray Spectroscopy (TEM-EDX), and Inductively Coupled Plasma Optical Emission Spectroscopy (ICP-OES) to quantify silver ionization. TEM confirmed spherical nanoparticles (5.54–61.46 nm), and FTIR validated *G. officinalis* functionalization on chitosan. AgChNPs with 1% chitosan at pH 4 exhibited optimal properties: a size of 207.88 nm, a zeta potential of +42.30 mV, and a PDI of 0.62. Biofilms displayed tunable mechanical strength, with a tensile strength of 3.48 MPa using 5% glycerol and 2% chitosan and an elongation at break of 24.99 mm. TGA showed a two-step degradation process (98.19% mass loss). Ag ionization was 62.57 mg/L in the liquid nanocomposite and 184.07 mg/kg in the biofilms. These findings highlight AgChNPs’ potential for controlled-release properties and enhanced mechanical performance, supporting sustainable agricultural applications.

## 1. Introduction

The current research area increasingly focuses on nanomaterials and nanostructures derived from physical, chemical, and biological sources, which play a pivotal role in advancing nanotechnology [1]. Metallic nanoparticles of various compositions have found widespread applications across industry, medicine, and agriculture [2,3,4,5]. Among these, silver nanoparticles (AgNPs) are the most extensively utilized nanomaterials, owing to their remarkable antibacterial, antiviral, antifungal, and insecticidal properties [6,7,8,9,10]. Their efficacy has been demonstrated in inducing mortality and developmental disruptions in various insect orders. For instance, AgNPs have shown larvicidal activity against *Spodoptera litura* (Lepidoptera: Noctuidae), strong adulticidal effects on *Callosobruchus maculatus* (Coleoptera: Chrysomelidae), and significant toxicity in *Musca domestica* (Diptera: Muscidae), while also reducing survival in aphids such as *Aphis nerii* (Hemiptera: Aphididae) [11,12,13,14]. These effects are primarily attributed to AgNP-induced oxidative stress caused by reactive oxygen species (ROS), membrane disruption, inhibition of key enzymatic systems, and interference with insect development and reproduction [15].

Traditionally, AgNPs are synthesized chemically through the reduction of silver salt solutions using reducing agents such as NaBH_4_, citrate, or ascorbate. However, these agents are often associated with environmental toxicity and biological hazards [16]. To address these concerns, green synthesis methods have gained prominence, leveraging biological systems and natural sources to eliminate toxic agents and minimize chemical inputs [17]. Plant-based synthesis, in particular, employs extracts, peels, fruits, rhizomes, and leaves containing bioactive compounds such as flavonoids, alkaloids, terpenoids, and polyphenols, which act as both reducing and stabilizing agents, facilitating the formation of AgNPs [18]. In this study, AgNPs were synthesized biologically using *Galega officinalis*, a perennial herbaceous species from the Fabaceae family. This plant was selected for its high total polyphenol and flavonoid content, which enhances its potential for nanoparticle synthesis. This approach offers a cost-effective, scalable, and environmentally friendly alternative, minimizing energy consumption and reducing reliance on toxic substances [19,20].

Stabilizers such as polymers have been extensively utilized to immobilize metal nanoparticles within matrix polymers. These stabilizers act as matrix materials, facilitating the control of nanoparticle growth and ensuring their stabilization [21]. Among these, Ch and its derivatives are natural cationic biopolymers widely recognized for their bioactivity, biocompatibility, and biodegradability [22]. Low-molecular weight Ch has the advantage of producing smaller and more uniform particle sizes [23]. in particular, Ch contains hydroxyl (-OH) and amino (-NH_2_) groups, which greatly stabilize the surface of AgNPs via electrostatic interactions to prevent their agglomeration [24]. Higher Ch concentrations enhance stability by providing greater coverage, forming a protective layer around AgNPs that controls silver ion release and ensures sustained ion liberation [25]. Therefore, the incorporation of Ch into a matrix of AgNPs has been shown to provide enhanced stability and reduced toxicity while mitigating oxidative corrosion [26,27].

Ch concentration and pH are critical parameters influencing AgNP stabilization and biofilm formation [28]. Moreover, slightly acidic pH conditions further promote denser biofilm formation by influencing Ch’s surface charge and its interactions with nanoparticles [29,30]. Glycerol, as a plasticizer in Ch-AgNP matrices, improves biofilm malleability and uniformity while regulating water vapor permeability and physical properties, thereby enhancing their role as protective barriers in food applications [31,32]. Beyond these structural functions, AgChNPs have demonstrated notable insecticidal properties. For instance, AgChNPs showed potent larvicidal and adulticidal activity against *Anopheles stephensi*, *Aedes aegypti*, and *Culex quinquefasciatus* (Diptera: Culicidae), with LC_50_ values ranging from 9.67 to 12.96 ppm depending on the developmental stage [33]. Similarly, AgChNPs tested against *Drosophila suzukii* (Diptera: Drosophilidae) caused mortality rates of up to 96% at 1000 ppm, significantly reduced adult emergence, and induced morphological abnormalities such as deformed wings and cuticle demelanization [34].

The objective of this study was to synthesize AgNPs coated with Ch (AgChNPs) by optimizing pH and Ch concentration to produce a stable colloidal nanocomposite. This optimized liquid nanocomposite was subsequently used to fabricate AgChNPs biofilms (bf-AgChNPs), with pH, glycerol, and Ch concentration as key reaction parameters. The resulting AgChNP biofilms were evaluated in terms of mechanical properties, biodegradability, and Ag^+^ release. These advancements aim to integrate AgChNP nanocomposites into sustainable agricultural strategies, particularly within the framework of the integrated pest management (IPM) approach.

## 2. Results and Discussion

### 2.1. Characterization of AgChNPs

#### 2.1.1. Visual Observation

The reduction of silver nitrate (Ag^+^) to metallic silver (Ag^0^) by the reducing agent *G. officinalis* was confirmed by a color change from light yellow to dark brown upon adjusting the pH to 11. This transition indicates the formation of colloidal biosynthesized AgNPs, characterized by the excitation of surface plasmon (SPR) vibrations [35]. Additionally, the color changes observed in AgNPs upon Ch coating further confirmed the formation of the nanocomposite, with variations ranging from light yellow at pH 3, dark brown at pH 4, to light brown at pH 5.

#### 2.1.2. UV-Vis Spectroscopy of AgChNPs

**Figure 1:**
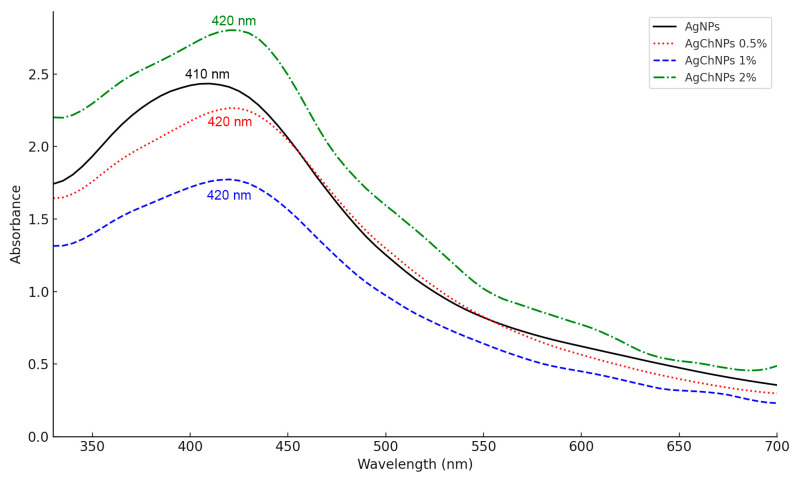
UV-Vis spectra of biosynthesized AgNPs (black), AgChNPs 0.5% (red), 1% (blue), 2% (green).

The characterization of AgChNPs using UV-vis spectroscopy was performed to identify the characteristic absorption peaks corresponding to the SPR of the AgChNP nanocomposite (Figure 1). The UV-vis spectrum of biosynthesized AgNPs without Ch exhibited a peak at 410 nm, consistent with findings by Zainab et al. [36], who reported a similar absorption peak for AgNPs synthesized via green methods using *Thermomyces lanuginosus*. For AgChNPs containing 0.5%, 1%, and 2% Ch, the nanocomposite displayed a peak at 420 nm, corresponding to the plasmon absorbance of AgNPs. The red shift observed in the AgNP peak following Ch treatment is attributed to an increase in the refractive index of the medium surrounding the metallic nanoparticles [37]. This observation aligns with reports by Rajeshkumar et al. [38], which identified SPR peaks in the range of 380–460 nm, confirming the successful formation of the AgChNP nanocomposite. Furthermore, Wulandari et al. [39] noted that SPR peaks within the 410–450 nm range are indicative of nanoparticles with a spherical morphology, although additional techniques such as TEM-EDX are required for definitive confirmation.

#### 2.1.3. Dynamic Light Scattering (DLS) and Zeta Potential of AgChNPs

**Figure 2:**
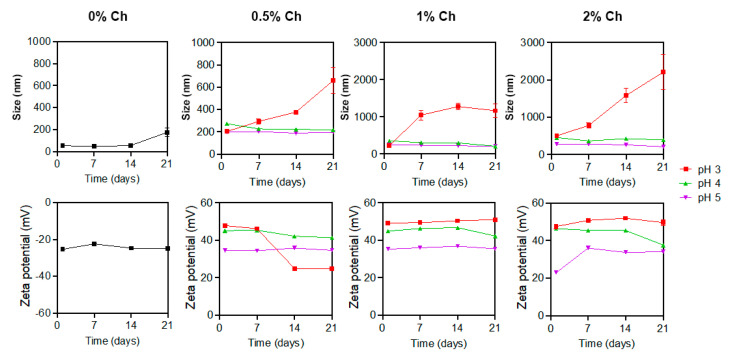
Changes in size and zeta potential of AgChNPs treated with 0.5 to 2% of Ch during 21 days of storage. Black line corresponds to AgNP 0% Ch treatment at pH 12 which was used as control.

The Z-average particle size (nm) and polydispersity index (PDI) of AgChNPs were determined using DLS measurements, while zeta potential results were used to assess particle stability (Figure 2). AgNPs synthesized using the *Galega officinalis* extract had an average size of 53.79 nm, which increased proportionally with the concentration of Ch used in the treatment. The zeta potential of AgNPs was negative at −25.37 mV, while after all Ch treatments, the AgChNPs turned positive (22.8 to 49.2 mV). The size increase and the change from negative to positive zeta potential were similarly observed in Ch-capped AgNPs synthesized using fungal extracts [40]. In the initial measurement, AgChNPs at 0.5% and pH 3 exhibited the smallest particle size (202.75 nm), followed by AgChNPs at 1% and pH 3, with a size of 224.80 nm. The latter demonstrated the highest zeta potential (+49.20 mV), showing significant differences compared to the control in both size and stability. For PDI, biosynthesized AgChNPs at 0.5% and pH 4 presented the lowest value (0.31), though no significant differences in PDI were observed among treatments or the control.

Subsequent measurements taken on days 7, 14, and 21 revealed significant increases in size for some AgChNP treatments. AgChNPs at 1% Ch and pH 3 showed a considerable size increase, exceeding the nanometric scale (1–1000 nm) and reaching 1165.65 nm by day 21. Similarly, treatments at 0.5% and 2% Ch and pH 3 exhibited significant size increases by day 21, measuring 658.70 nm and 2209.23 nm, respectively, with the highest values showing significant differences compared to the control.

In contrast, other treatments demonstrated greater stability. AgChNPs at 1% Ch and pH 4 exhibited a decrease in size over time, reaching 207.88 nm on day 21, with values approaching those of the control, which consisted of biosynthesized AgNPs. The control size increased from 53.79 nm to 175.23 nm over the 21 days, with no significant differences observed between the control and AgChNPs at 1% Ch and pH 4. The smallest particle size was observed in AgChNPs at 0.5% Ch and pH 5, measuring 196.33 nm on day 21. However, this treatment exhibited a zeta potential value of +34.73 mV, which indicates good stability and reduced aggregation of the nanoparticles in the dispersion. Generally, a reduction in pH increases the zeta potential of AgChNPs due to the protonation of amino groups in the Ch molecules. Below the pKa of the amino groups (6.0–6.5), these groups become protonated, resulting in a positive charge on the nanoparticles [41].

Regarding zeta potential, after 21 days (Figure 2), AgChNPs at 1% and 2% Ch and pH 3 exhibited the highest values, with +51.03 mV and +39.57 mV, respectively. However, both treatments displayed particle sizes exceeding the nanometric scale (>1000 nm), indicating aggregation and low long-term stability of the AgNPs, which limits their suitability for certain applications. The most favorable treatment in terms of zeta potential and particle size was AgChNPs at 1% Ch and pH 4, with a zeta potential of +42.30 mV.

The effect of reaction parameters on the PDI is summarized in Table 1. After 21 days, the lowest PDI values were observed in AgChNPs at 0.5% and 1% Ch and pH 5, both with a PDI of 0.44. However, no significant differences were noted between these treatments and the control, indicating that the nanoparticles were monodispersed and free from aggregation. Based on the overall findings, the optimal conditions for the biosynthesis of AgChNPs were identified as 1% Ch at pH 4, yielding an average particle size of 207.88 nm, a zeta potential of +42.30 mV, and a PDI of 0.62. This nanocomposite was selected for further characterization via FTIR, XRD, and TEM-EDX analysis.

The optimization of the AgChNP biosynthesis process demonstrated that both pH and Ch concentration significantly influence particle size distribution and zeta potential. These findings are consistent with those of Karuppaiah et al. [42], who reported an increase in nanoparticle size after Ch coating (545 nm), resulting in monodisperse nanoparticles (PDI of 0.26) and a shift in zeta potential from negative (−36.3 mV) for biosynthesized AgNPs to positive (+37.5 mV) upon Ch addition. This shift suggests that the Ch shell imparts a positive surface charge, leading to a positive zeta potential. Similarly, Kanniah et al. [43] highlighted that biopolymer-stabilized metallic nanoparticles are more effective against pH and electrolyte fluctuations compared to chemical stabilizers.

Mbae and Umesha [44] further noted that the degree of deacetylation and concentration of Ch influence the number of ionizable groups on Ch chains coating the nanoparticles, contributing to higher positive zeta potential values (+26 mV). Ch adopts various conformations on the nanoparticle surface, enhancing stabilization. Barbosa et al. [45] reported that medium molecular weight Ch exhibits a zeta potential of approximately +20 mV at neutral pH. However, at lower pH levels (2–4) and concentrations above 0.3% (*w*/*v*), the acidic moieties of the polymeric chains become unionized, increasing hydrophobicity due to interactions with hydronium ions (H_3_O^+^), which accumulate at the interface between water and hydrophobic media.

This phenomenon aligns with the considerable size increases observed for AgChNPs at pH 3 in this study’s DLS measurements. At higher Ch concentrations (0.5%, 1%, and 2%), the hydrophobic effect appears to diminish, likely due to structural and concentration-related changes in Ch conformations, which further influence nanoparticle stability and size.

#### 2.1.4. Fourier-Transform Infrared Spectroscopy (FTIR) of AgChNPs

**Figure 3:**
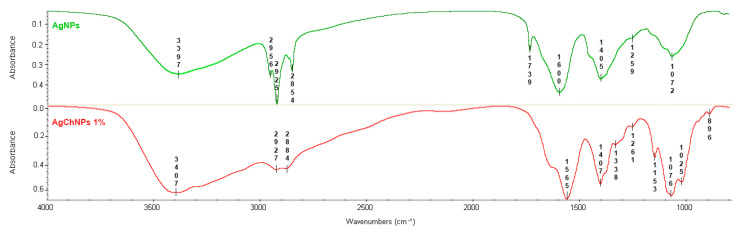
FTIR spectrum of biosynthesized AgNPs (green) and AgChNPs 1% pH 4 (red).

The FTIR analysis identified biomolecules in *G. officinalis* leaf extract and Ch that contribute to silver ion (Ag^+^) reduction, nanoparticle stabilization, and interactions between Ch and biosynthesized AgNPs (Figure 3). The FTIR spectra of AgNPs without Ch exhibited characteristic absorption bands at 3397 cm^−1^ (-OH groups), 2956 cm^−1^ (amine salt stretching), and 2925 cm^−1^ and 2854 cm^−1^ (C-H stretching) [46,47,48]. Peaks at 1739 cm^−1^, 1600 cm^−1^, 1405 cm^−1^, 1259 cm^−1^, and 1072 cm^−1^ corresponded to C=O esters/acids, aromatic C=C, C-N aliphatic amines, and C-O stretching vibrations of alcohols [49,50,51].

For AgChNPs, the FTIR spectrum revealed prominent bands at 3407 cm^−1^ (O-H/NH_2_ stretching), 2956 cm^−1^ (C-H stretching), and 2927 cm^−1^ and 2884 cm^−1^ (CH_2_ stretching) [46]. Peaks at 1565 cm^−1^ and 1338 cm^−1^, along with bands between 1407–1025 cm^−1^, corresponded to amide I (C=O stretching), amide III (C-N stretching), and CH_3_ and C-O-C bending vibrations, indicating the presence of carbohydrate structures [52,53]. Additionally, the absorption band at 896 cm^−1^ confirmed the polysaccharide structure of Ch, consistent with findings by Malakhovska et al. [54], who identified C-H deformation bands as markers of polysaccharide-nanoparticle interactions.

A slight shift in the O-H/NH_2_ band from 3396 cm^−1^ in AgNPs to 3407 cm^−1^ in AgChNPs suggests coordination between AgNPs and the functional groups of Ch, contributing to nanocomposite stabilization. This interaction highlights the role of the NH_2_ group in metal ion chelation [55]. Moreover, distinct differences were observed in the spectral region between 1000 and 1100 cm^−1^. While AgNPs exhibited a single peak at 1072 cm^−1^, AgChNPs displayed two well-resolved bands at 1026 cm^−1^ and 1076 cm^−1^ [25]. These bands are typically attributed to the stretching vibrations of C–O–C and C–O bonds in polysaccharides such as Ch [56]. The presence of two distinct peaks in AgChNPs, compared to the single band in AgNPs, suggests a greater contribution of these vibrational modes due to the Ch coating, providing compelling evidence of its successful integration onto the nanoparticle surface [57]. This chemical modification reflects the formation of a Ch-nanoparticle interface [58]. Therefore, the spectral features between 1000 and 1100 cm^−1^ serve as reliable indicators of the presence of Ch on the AgNP surface, reinforcing its role in enhancing nanoparticle stability and dispersion within the polymer matrix [59].

#### 2.1.5. X-Ray Diffraction (XRD) Analysis of AgChNPs

**Figure 4:**
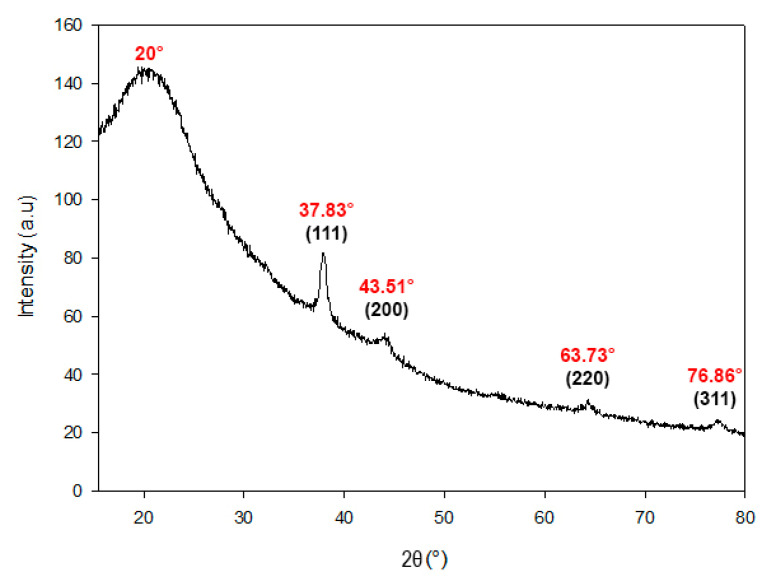
The XRD pattern of biosynthesized AgChNPs (1% Ch, pH 4). The XRD peaks correspond to the crystallographic planes (111), (200), (220), and (311) of the face-centered cubic (FCC) structure of metallic silver.

The XRD analysis was conducted to determine the crystalline structure of silver (Ag) in the biosynthesized nanocomposite. The XRD patterns revealed distinct peaks corresponding to silver crystals, confirming the presence of Ag^0^ in the AgChNPs (Figure 4). Specifically, diffraction peaks were observed at 37,83°, 43,51°, 63,73°, and 76,86°, which correspond to the crystallographic planes (111), (200), (220), and (311) of the face-centered cubic (FCC) structure of metallic silver. A broad diffraction hump centered at 2θ = 20° was also observed, which may be attributed to the amorphous scattering of Ch chains, consistent with previously reported patterns for semi-crystalline biopolymers [60,61].

These results align with Naqvi et al. [62], who reported intense peaks at 38.26°, 44.37°, 65.41°, and 77.29°, indicating the FCC structure of AgNPs synthesized from *Juglans regia* extract. Similarly, Kumari et al. [63] observed peaks at 37.8°, 43.4°, 63.2°, and 76.6° in AgNPs synthesized from *Euphorbia royleana* extract, further supporting the FCC structure of biosynthesized AgNPs.

#### 2.1.6. Transmission Electron Microscopy (TEM-EDX) Analysis of AgChNPs

**Figure 5:**
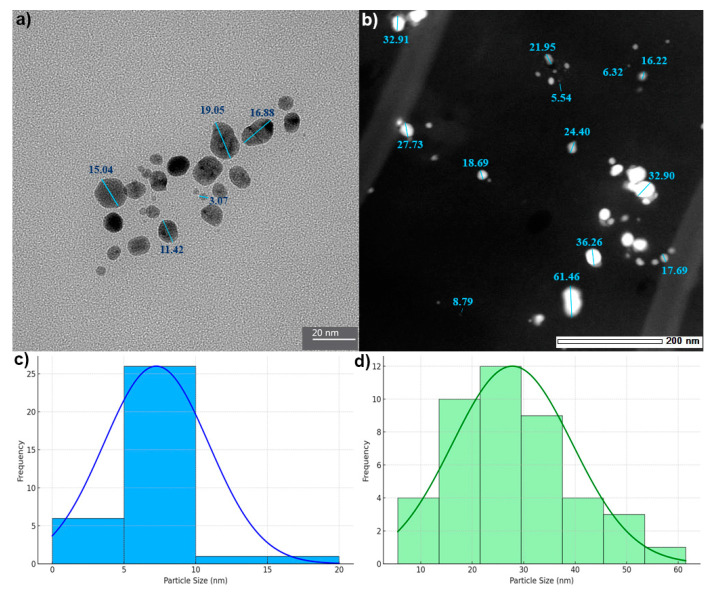
Transmission electron microscopy (TEM) characterization and particle size distribution of silver-based nanomaterials. (**a**) TEM image of biosynthesized AgNPs with sizes ranging from 3.07 to 19.05 nm; (**b**) TEM image of biosynthesized AgChNPs with sizes ranging from 5.54 to 61.46 nm; (**c**) Histogram of AgNP particle size distribution derived from TEM measurements; (**d**) Histogram of AgChNP particle size distribution.

TEM was employed to assess the morphology and size of biosynthesized AgNPs and AgChNPs. AgNPs reduced by *Galega officinalis* extract (Figure 5a) appeared predominantly spherical, with a narrow size range of 3.07–19.05 nm. These values differed from DLS measurements, which reported sizes between 53.79 and 175.23 nm over 21 days (PDI = 0.52), indicating moderate polydispersity in the colloidal suspension.

Similarly, AgChNPs exhibited spherical morphology in TEM (Figure 5b), with diameters ranging from 5.5 to 61.46 nm, while DLS indicated a larger hydrodynamic diameter of 207.88 nm and a higher PDI of 0.62, suggesting increased polydispersity. These differences reflect the methodological contrast between TEM and DLS. TEM measures dehydrated core sizes, whereas DLS accounts for solvation layers, surface-bound biomolecules, and particle aggregation in solution [64,65]. These findings are consistent with those of Azimi et al. [66], who observed ~25 nm spherical AgNPs using *G. officinalis* via TEM. Variations in synthesis conditions, such as extract preparation, Ag^+^ concentration, pH, and temperature, likely explain the size differences between studies [67,68].

Regarding AgChNPs, Martínez-Cisterna et al. [34] reported a similar trend, with TEM diameters ranging from 2.8 to 65.3 nm and DLS values up to 257.2 nm, highlighting nanoparticle aggregation in suspension. Affes et al. [69] showed that low-molecular weight Ch promotes the formation of ~5 nm AgNPs, supporting the stabilizing role of biopolymers. Wulandari et al. [39] also noted size ranges of 51–255 nm (for AgNPs) and 55–371 nm (for AgChNPs), with DLS consistently reporting larger sizes than SEM due to drying-induced clustering, while better reflecting dispersion behavior.

**Figure 6:**
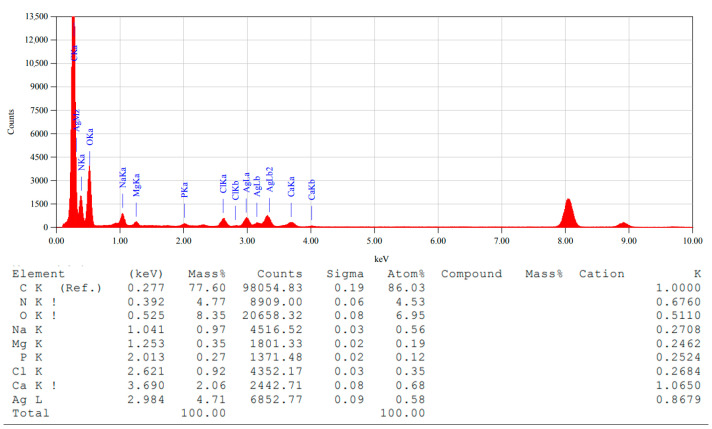
Energy dispersive X-ray (EDX) spectrum of biosynthesized AgChNPs (1% Ch, pH 4).

Energy Dispersive X-ray (EDX) spectroscopy was conducted to validate the elemental composition of the biosynthesized AgChNPs (Figure 6). A strong Ag signal was observed at 2.98 keV, corresponding to a silver mass percentage of 4.71%. This result is consistent with previous studies that report metallic silver nanocrystals exhibiting characteristic absorption spectra within the 2.4–4.0 keV range [70].

In addition to Ag, peaks for elements such as C, N, O, Na, Mg, P, Cl, and Ca were detected. These elements can be attributed to the phytochemicals present in the *G. officinalis* leaf extract and Ch composition. This finding aligns with Wang et al. [71], who also reported the presence of C and O in AgNPs synthesized using a Ch-*Mentha piperita* biocomposite. The detection of these additional elements suggests that *G. officinalis* extract serves a dual role as both a reducing and stabilizing agent during nanoparticle synthesis, enhancing the biocompatibility and functional diversity of AgChNPs.

### 2.2. Biosynthesis of Ch-Coated Silver Nanoparticle Biofilm (bf-AgChNPs)

#### 2.2.1. Physical, Structural, and Mechanical Properties Characterization Biofilms

Biofilm thickness measurements (Table 2) showed no significant differences among measurable treatments; however, treatments with low Ch concentrations (0.5% or none) and the control group exhibited insufficient structural integrity. These findings emphasize Ch’s role in biofilm stability. For example, Santos et al. [37] observed that Ch concentrations below 1% result in fragile, discontinuous films due to weaker ionic bonding and intermolecular interactions. Similarly, Haghighi et al. [72] demonstrated that 1–2% Ch promotes the formation of robust three-dimensional matrices with cross-linked networks.

Mechanical property evaluations (Table 2) revealed that the bf-glychph-5-02-05 treatment achieved the highest tensile strength (TS) at 3.48 MPa, while the bf-glychph-5-02-04 treatment exhibited the greatest elongation at break (EAB) at 24.99 mm. These results highlight the synergy between 5% glycerol and 2% Ch, which optimized both TS and EAB. This is consistent with the findings presented by Pava et al. [73], who reported that glycerol enhances EAB by reducing hydrogen bonding while maintaining a balance with TS.

The significant impact of Ch on TS is attributed to its ability to form strong intermolecular interactions [74]. Additionally, the incorporation of AgNPs reinforced the polymer matrix, enhancing mechanical properties and maintaining flexibility through uniform nanoparticle dispersion, contributing to biofilm stability and barrier properties, potentially extending biofilm shelf life [31,75].

#### 2.2.2. Thermal Stability Analysis of AgChNP Biofilms

**Figure 7:**
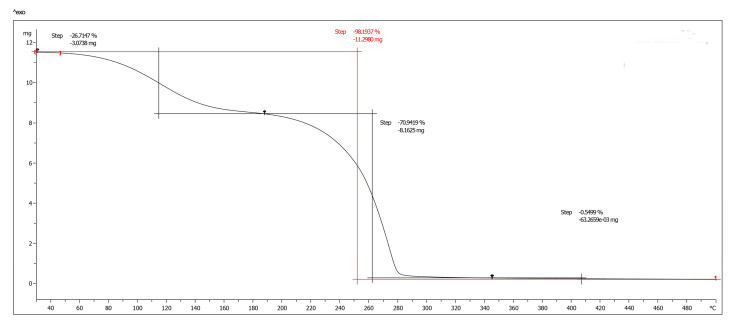
Thermogravimetric analysis (TGA) of AgChNP biofilm at 15% glycerol and 2% Ch concentrations and pH 4.

Thermogravimetric analysis (TGA) was performed to evaluate the thermal stability and decomposition behavior of biofilms containing 15% glycerol and 2% Ch at pH 4 (Figure 7). Two major mass loss events were observed from an initial sample mass of 11.50 mg. The first event, occurring between 40 °C and 190 °C, accounted for a 26.71% loss (−3.07 mg), likely due to the evaporation of residual moisture and volatile compounds. The second event, occurring between 190 °C and 280 °C, resulted in a further 70.94% loss (−8.16 mg), culminating in a total mass loss of 98.19% (−11.30 mg).

This two-step degradation pattern is characteristic of polymeric and composite materials, where moisture loss is followed by the thermal decomposition of the polymer matrix at elevated temperatures. Basavegowda and Baek [76] emphasized the environmental benefits and biodegradability of biopolymer-based nanocomposites compared to synthetic polymers. The significant mass loss observed above 240 °C in this study supports the predominance of biodegradable organic components within the biofilms.

These findings highlight the dual advantages of the evaluated nanocomposites: thermal stability suitable for targeted applications and environmentally friendly degradation, making them promising candidates for sustainable end-of-life applications.

#### 2.2.3. Release of Ag^+^ from AgChNP Nanocomposites

**Figure 8:**
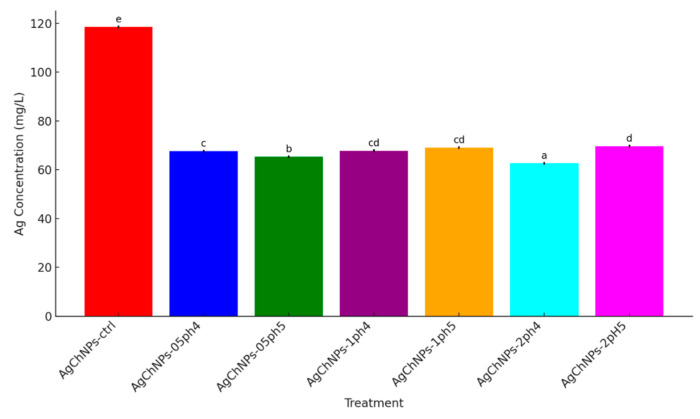
Silver concentration (mg/L) in biosynthesized AgChNP liquid nanocomposites. The figure displays the concentration of silver (Ag) in solution for different AgChNP treatments (0.5%, 1%, and 2% Ch, pH 3, 4 and 5). The letters indicated in the figure identifies statistically significant differences between treatment groups (Duncan’s HSD test).

The release of metal ions from nanomaterials refers to the process by which ions are liberated from the surface or core of nanoparticles into the surrounding medium. This typically occurs due to chemical or physical interactions, including surface oxidation, dissolution, or nanoparticle degradation [77]. In this study, the concentration of silver ions (Ag^+^) released from AgChNP nanocomposites varied significantly depending on the pH level and Ch concentration (Figure 8). The control treatment (AgNPs-ctrl) exhibited the highest Ag^+^ concentration in solution (118.60 mg/L), while the AgChNPs 2%, pH 4 treatment displayed the lowest concentration (62.57 mg/L), suggesting enhanced nanoparticle stabilization under high Ch content and acidic conditions.

Intermediate Ag^+^ levels were observed in AgChNPs 2% at pH 5 (69.60 mg/L), likely due to increased Ag solubility and weakened electrostatic interactions at higher pH values. Similarly, AgChNPs 0.5% at pH 4 and pH 5 showed Ag^+^ concentrations of 67.63 mg/L and 65.42 mg/L, respectively, indicating that lower Ch concentrations provide moderate stabilization. Treatments containing 1% Ch exhibited minimal variation between pH conditions (67.79 mg/L at pH 4 and 69.09 mg/L at pH 5), supporting the role of Ch as a stabilizing agent across different environments. These observations are in agreement with findings by Nasef et al. [78], who reported that Ch protonation at acidic pH enhances electrostatic interactions, thereby reducing Ag^+^ release.

**Figure 9:**
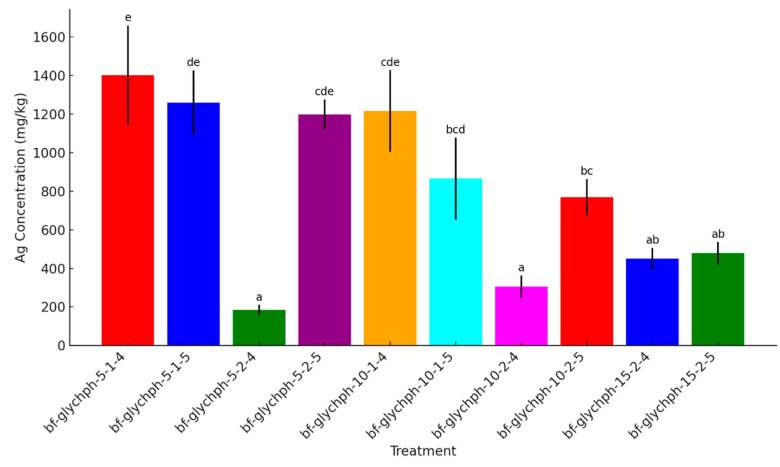
Silver (Ag) concentrations in different AgChNP nanocomposite biofilms. The figure displays the concentration of silver (Ag) in AgChNP biofilm treatments (0.5%, 1%, and 2% Ch, 5%, 10%, 15% glycerol, pH 3, 4 and 5). The letters indicated in the figure identifies statistically significant differences between treatment groups (Duncan’s HSD test).

In the case of biofilm nanocomposites (Figure 9), significant differences in Ag^+^ release were also observed. For instance, the sample bf-glychph-5-1-4 exhibited the highest Ag^+^ content (1403.00 mg/kg), while bf-glychph-5-2-4 released significantly less (184.07 mg/kg), likely due to pH-dependent interactions between Ch and AgNPs. These results are consistent with previous findings showing enhanced Ag^+^ binding to protonated Ch at lower pH, which reduces ion diffusion [79]. Similarly, samples bf-glychph-10-2-4 and bf-glychph-15-2-4 released 304.03 mg/kg and 449.93 mg/kg of Ag^+^, respectively, indicating that increasing Ch concentration contributes to matrix robustness and improved film-forming properties.

Compared to conventional AgNPs, which release Ag^+^ due to surface oxidation and limited colloidal stability, AgChNPs exhibited a more controlled and sustained release profile under similar conditions. This behavior is primarily attributed to the Ch coating, which forms a semi-permeable polyelectrolyte matrix that modulates ion diffusion and enhances particle stability [80]. The pH-responsive release observed supports the role of protonated Ch in strengthening electrostatic interactions with AgNPs, particularly under acidic conditions, thereby reducing Ag^+^ leaching [78]. Furthermore, the Ch matrix acts both as a physical barrier and as an active chelating agent, with amino (–NH_2_) and hydroxyl (–OH) groups capable of coordinating Ag^+^ ions and limiting their mobility [79]. This mechanism is especially relevant in biofilm formulations, where Ch concentrations above 2% enhance structural cohesion and reduce early-phase ion release. Similar findings have been reported in other AgChNP systems, where reduced Ag^+^ release was linked to strong ion binding and slow diffusion through a compact matrix [81].

Finally, Ag^+^ release occurred under all tested pH conditions, with the lowest concentrations detected at pH 4, indicating enhanced nanoparticle stability in acidic environments. These features collectively position AgChNPs as promising candidates for pH-sensitive applications, including crop protection films.

## 3. Materials and Methods

### 3.1. Plant Leaves and Chemical Obtention

Dried *Galega officinalis* leaves were sourced from Botanic Universe (Burlington, ON, Canada) and processed into small, sifted pieces. Silver nitrate (AgNO_3_) and low-molecular weight Ch (50–190 kDa; deacetylation degree: > 75%) were purchased from Sigma-Aldrich (Canada Co, Oakville, ON, Canada).

### 3.2. Biosynthesis of AgNPs (AgNPs)

The AgNPs were synthesized following the methodology of Manosalva et al. [19]. To prepare the extract, 10 g of dried *G. officinalis* leaves were placed in a 250 mL Erlenmeyer flask with 100 mL of ultrapure water (milli-q) and boiled at 100 °C for 5 min. The extract was then cooled to room temperature (25 ± 2 °C), diluted with 10% (*v*/*v*) ultrapure water, and filtered using a vacuum pump with Whatman No. 1 filter paper.

A concentrated AgNO_3_ stock solution (100 mM) was added to the *G. officinalis* extract to achieve a final silver ion concentration of 1.5 mM, and the pH was adjusted to 11 using 1 M NaOH. The reaction was maintained at room temperature (25 ± 2 °C) under continuous stirring for 24 h on a magnetic stirrer. The resulting sample was frozen at −80 °C and lyophilized for characterization and bioassays.

The same AgNP synthesis parameters were consistently applied across all assays.

### 3.3. Biosynthesis of Ch-Coated AgNPs (AgChNPs)

The biosynthesized AgNPs were treated with Ch following a modified methodology from Wulandari et al. [39]. Briefly, AgNP dispersion and Ch solutions were mixed in a 1:1 volume ratio, with Ch concentrations of 0.5%, 1%, and 2% (prepared in 2% acetic acid). The mixtures were subjected to varying pH conditions (3, 4, and 5), which were adjusted using NaOH and HCl. The resulting AgChNPs were homogenized on a magnetic stirrer at 800 rpm for 24 h. AgNPs without Ch were used as the control.

### 3.4. Characterization of Ch-Coated AgNPs (AgChNPs)

#### 3.4.1. Dynamic Light Scattering (DLS) and Zeta Potential

The particle size distribution of the AgChNPs was determined using a Zetasizer Nano Zs (Malvern Instruments Ltd., Malvern, UK). Measurements included the average hydrodynamic diameter (nm), PDI, and zeta potential (mV). These parameters were evaluated at 7-day intervals over a 21-day period to assess the size, polydispersity, and stability of the nanoparticles over time. The nanocomposite exhibiting optimal stability and uniformity was selected for further characterization using FTIR, XRD, and TEM-EDX analysis.

#### 3.4.2. UV-Vis Spectroscopy

The formation of biosynthesized AgNPs and their incorporation into AgChNPs were monitored using a SpectraMax UV-vis spectrophotometer (Molecular Devices, San Jose, CA, USA). Absorption spectra were recorded in the range of 300 to 800 nm, with ultrapure water serving as the blank.

#### 3.4.3. FTIR Spectroscopy

The AgChNPs were characterized using FTIR spectroscopy to identify functional groups before and after Ch coating. Spectra were recorded in the range of 500–4000 cm^−1^ using a Nicolet 6700 FT-IR spectrometer (Thermo Fisher Scientific, Waltham, MA, USA).

#### 3.4.4. XRD Analysis

The crystalline structure of the biosynthesized AgChNPs was analyzed using XRD. Measurements were performed with a Bruker D8 Advance X-ray diffractometer (Bruker AXS GmbH, Karlsruhe, Germany) equipped with Cu-Kα radiation (K = 1.5406 Å) at 30 kV and 40 mA, over a 2θ range of 20–100°.

#### 3.4.5. Transmission Electron Microscopy Coupled with Energy Dispersive X-Ray Spectroscopy (TEM-EDX)

Transmission electron microscopy coupled with energy dispersive X-ray spectroscopy (TEM-EDX) was performed using a JEOL JEM-ARM200CF S/TEM equipped with an EDX system (JEOL Ltd., Tokyo, Japan) to confirm the formation, size, and morphology of AgChNPs, as well as to determine the elemental composition and mass percentage within the nanocomposite.

### 3.5. Biosynthesis of Ch-Coated Silver Nanoparticle Biofilm

The biofilms were prepared using AgChNPs treated with varying Ch concentrations and adjusted to pH levels ranging from 3 to 5, as described in Activity 2.3. These were blended with glycerol at concentrations of 5%, 10%, and 15% (*v*/*v*) as a plasticizing agent, following an adaptation of the methodology by Affes et al. [69]. The mixtures were stirred on a magnetic stirrer for 30 min to ensure homogeneity. The resulting biofilm mixtures were then poured into plastic petri dishes (5.2 cm in diameter) and dried at room temperature (25 ± 2 °C) for 48 h to allow complete solvent evaporation. The dried films were carefully peeled from the dish surfaces and stored at 25 °C with a relative humidity of 50%.

### 3.6. Physical and Structural Characterization of Ch-Coated Silver Nanoparticle Biofilm

#### 3.6.1. Films Thickness

The thickness of the films was measured using a Vogel high-precision external micrometer (DIN 863). Measurements were taken at three random locations on each film sample, and the average thickness was calculated and used for further analysis.

#### 3.6.2. Mechanical Properties

The mechanical properties of nanocomposite biofilms, including tensile strength (TS, MPa) and elongation at break (EAB, %), were assessed using an Instron testing instrument. Rectangular film samples (1.0 × 4.0 cm) were conditioned at 25 °C and 50% RH for seven days prior to testing. Measurements were conducted at a deformation rate of 5 mm/min in accordance with ISO standards (ISO, 1995). Film thickness was measured, and tests were performed at 25 ± 2 °C, with three replicates per formulation.

### 3.7. Thermal Stability Analysis

The biodegradability of the biofilms was assessed using thermogravimetric analysis (TGA) on a TA Discovery TGA instrument. Samples were heated from 30 °C to 500 °C at a rate of 20 °C/min under a nitrogen atmosphere. Weight loss (Δw), the temperature at the maximum degradation rate (Tmax), and final residue values were evaluated using STARe SW 16.10 software. The analysis focused on AgChNPs formulated with 15% glycerol, 2% Ch, and pH 4, which were selected for their enhanced stability.

### 3.8. Inductively Coupled Plasma Atomic of Biofilm (ICP-OES)

The Ag^+^ concentration in both liquid and biofilm nanocomposites, before and after Ch coating, was quantified using an inductively coupled plasma-optical emission spectrometer (ICP-OES, Thermo iCAP6300 Duo, Thermo Fisher Scientific, Waltham, MA, USA). Samples were atomized in an argon plasma (5500–8000 K), and analyte emissions were detected using yttrium (Y) as an internal standard. Certified multi-element standard solutions ensured calibration, and external reference standards validated the results. Uncoated AgNPs served as controls to establish baseline silver ion release. The method adhered to EPA 200.7 and EPA 6010d standards, ensuring precision and reproducibility.

### 3.9. Statistical Analysis

All experiments were conducted in triplicate, and the mean values are reported. Statistical analyses were performed using one-way analysis of variance (ANOVA) in SPSS software (version 20.0). Post hoc comparisons among group means were conducted using Duncan’s HSD test, with a significance level set at *p* < 0.05.

## 4. Conclusions

In conclusion, the present study demonstrates that AgChNPs exhibit controlled and pH-dependent Ag^+^ ion release, which is enhanced by higher Ch concentrations and acidic conditions. These properties confer distinct advantages over conventional AgNPs, including improved colloidal stability and sustained release kinetics, which are modulated by both matrix density and environmental pH. The dual capacity of Ch to form a mechanical diffusion barrier and to chelate silver ions explains the significant reduction in Ag^+^ liberation observed in both colloidal and biofilm formulations.

The incorporation of Ch also significantly improved mechanical properties, such as tensile strength and elongation at break, while enabling controlled silver ion release. In addition, thermogravimetric analysis (TGA) confirmed the thermal stability and biodegradability of these nanocomposites, supporting their suitability for environmentally sustainable applications.

Beyond physicochemical performance, AgChNPs exhibit considerable biological potential. Recent studies have highlighted the efficacy of AgNPs as nanopesticides, with documented toxicity against agriculturally important insect orders. These nanomaterials act through multiple mechanisms, including disruption of enzymatic systems, induction of oxidative stress, and interference with insect development, showing efficacy levels comparable to commercial pesticides. In this context, AgChNPs represent a promising alternative for agricultural implementation, where their pH-responsive release behavior could be harnessed in crop protection matrices (e.g., films, sprays, or coatings) that ensure targeted delivery and reduced environmental impact as part of IPM strategies. Future research should focus on validating these systems under field conditions and evaluating their interactions with crops, pests, and non-target organisms to fully elucidate their benefits and potential ecological risks.

## Figures and Tables

**Table 1:** Polydispersity index (PDI) of biosynthesized AgNPs and AgChNPs at 0.5%, 1% and 2%, Ch concentrations at different pH levels over 21 days.

Ch Concentration (%)	pH	PDI			
		Day 1	Day 7	Day 14	Day 21
0.5	3	0.53 ± 0.14	0.39 ± 0.04	0.27 ± 0.08	0.68 ± 0.10
	4	0.31 ± 0.02	0.43 ± 0.09	0.36 ± 0.05	0.45 ± 0.12
	5	0.42 ± 0.10	0.44 ± 0.11	0.43 ± 0.12	0.44 ± 0.11
1	3	0.48 ± 0.12	0.79 ± 0.10	0.69 ± 0.04	0.67 ± 0.15
	4	0.41 ± 0.05	0.39 ± 0.06	0.39 ± 0.06	0.62 ± 0.16
	5	0.38 ± 0.07	0.37 ± 0.06	0.46 ± 0.12	0.44 ± 0.12
2	3	0.46 ± 0.07	0.80 ± 0.07	0.84 ± 0.10	0.75 ± 0.11
	4	0.48 ± 0.09	0.54 ± 0.12	0.40 ± 0.05	0.41 ± 0.07
	5	0.52 ± 0.12	0.50 ± 0.12	0.49 ± 0.12	0.55 ± 0.15
0	12	0.66 ± 0.15	0.68 ± 0.14	0.71 ± 0.13	0.52 ± 0.01

The absence of letters is due to the lack of significant differences.

**Table 2:** Thickness, load at tensile strength and extension at break of AgChNP biofilms.

Biofilm Sample				Biofilm Characteristics		
Glycerol (%)	Ch (%)	pH	Assigned Label	Thickness (mm)	Load at Tensile Strength (N)	Extension at Break (mm)
5	0.5	4	bf-glychph-5-05-4	nbf	nbf	nbf
	0.5	5	bf-glychph-5-05-5	nbf	nbf	nbf
	1.0	4	bf-glychph-5-1-4	0.23 ± 0.04 ^a^	0.80 ± 0.23 ^a^	6.29 ± 1.54 ^a^
	1.0	5	bf-glychph-5-1-5	0.28 ± 0.05 ^ab^	1.41 ± 0.01 ^ab^	16.05 ± 1.51 ^ab^
	2.0	4	bf-glychph-5-2-4	0.23 ± 0.02 ^a^	2.94 ± 0.50 ^cd^	24.99 ± 3.62 ^b^
	2.0	5	bf-glychph-5-2-5	0.25 ± 0.05 ^ab^	3.48 ± 0.06 ^d^	15.68 ± 1.86 ^ab^
10	0.5	4	bf-glychph-10-05-4	nbf	nbf	nbf
	0.5	5	bf-glychph-10-05-5	nbf	nbf	nbf
	1.0	4	bf-glychph-10-1-4	0.25 ± 0.03 ^ab^	0.71 ± 0.23 ^a^	6.80 ± 1.32 ^a^
	1.0	5	bf-glychph-10-1-5	0.23 ± 0.04 ^a^	0.97 ± 0.12 ^ab^	10.59 ± 2.31 ^a^
	2.0	4	bf-glychph-10-2-4	0.50 ± 0.07 ^ab^	2.16 ± 0.10 ^bc^	11.82 ± 2.16 ^a^
	2.0	5	bf-glychph-10-2-5	0.28 ± 0.01 ^ab^	2.75 ± 0.38 ^cd^	12.65 ± 2.67 ^a^
15	0.5	4	bf-glychph-15-05-4	nbf	nbf	nbf
	0.5	4	bf-glychph-15-05-5	nbf	nbf	nbf
	1.0	4	bf-glychph-15-1-4	nbf	nbf	nbf
	1.0	5	bf-glychph-15-1-5	nbf	nbf	nbf
	2.0	4	bf-glychph-15-2-4	0.52 ± 0.12 ^a^	1.09 ± 0.15 ^ab^	12.73 ± 2.79 ^a^
	2.0	5	bf-glychph-15-2-5	0.39 ± 0.05 ^ab^	1.32 ± 0.25 ^ab^	8.33 ± 097 ^a^

Within each column, mean ± SD followed by the same letters do not differ significantly (ANOVA, Duncan HSD test, *p* ≥ 0.05). nbf: indicates no biofilm formation.

## Data Availability

Data are contained within the article.
