# Correction: Martínez-Cisterna et al. Chitosan-Coated Silver Nanocomposites: Biosynthesis, Mechanical Properties, and Ag^+^ Release in Liquid and Film Forms. *Int. J. Mol. Sci.* 2025, *26*, 4130

**DOI:** 10.3390/ijms27167326

**Published:** 2026-08-17

**Authors:** Daniel Martínez-Cisterna, Lingyun Chen, Leonardo Bardehle, Edward Hermosilla, Gonzalo Tortella, Manuel Chacón-Fuentes, Olga Rubilar

**Affiliations:** 1 Doctorado en Ciencias de Recursos Naturales, Facultad de Ciencias Químicas y Recursos Naturales, Universidad de La Frontera, Av. Francisco Salazar 01145, Casilla 54-D, Temuco 4811230, Chile; d.martinez11@ufromail.cl; 2 Centro de Investigación Biotecnológica Aplicada al Medio Ambiente (CIBAMA), Universidad de La Frontera, Av. Francisco Salazar 01145, Casilla 54-D, Temuco 4811230, Chile; leonardo.bardehle@ufrontera.cl (L.B.); edward.hermosilla@ufrontera.cl (E.H.); gonzalo.tortella@ufrontera.cl (G.T.); 3 Laboratorio de Química Ecológica, Departamento de Ciencias Químicas y Recursos Naturales, Universidad de La Frontera, Av. Francisco Salazar 01145, Casilla 54-D, Temuco 4811230, Chile; 4 Department of Chemical Engineering, Universidad de La Frontera, Av. Francisco Salazar 01145, Temuco 4811230, Chile; 5 Department of Agriculture, Food and Nutritional Sciences, University of Alberta, Edmonton, AB T6G 2P5, Canada; 6 Departamento de Producción Agropecuaria, Facultad de Ciencias Agropecuarias y Medioambiente, Universidad de La Frontera, Av. Francisco Salazar 01145, Casilla 54-D, Temuco 4811230, Chile; 7 Agriaquaculture Nutritional Genomic Center, CGNA, Temuco 4780000, Chile; manuel.chacon@cgna.cl

The authors would like to make the following corrections to their published paper [1]. The changes are as follows:

## Reference Correction

There was a citation error in the original publication [1]. Reference [50] cited in the published manuscript was retracted by the publisher in December 2025.

50. Muthukumaran, U.; Govindarajan, M.; Rajeswary, M.; Hoti, S.L. Synthesis and characterization of silver nanoparticles using Gmelina asiatica leaf extract against filariasis, dengue, and malaria vector mosquitoes. *Parasitol. Res.* **2015**, *114*, 1817–1827. https://doi.org/10.1007/s00436-015-4368-4.

To uphold the highest standards of academic integrity and ensure the accuracy of the scientific record, the authors decided to remove reference [50].

A correction has been made to the Results and Discussion, Section 2.1.4:

Peaks at 1739 cm^−1^, 1600 cm^−1^, 1405 cm^−1^, 1259 cm^−1^, and 1072 cm^−1^ corresponded to C=O esters/acids, aromatic C=C, C-N aliphatic amines, and C-O stretching vibrations of alcohols [49,50].

With this correction, the order of some references has been adjusted accordingly.

## Text Correction

In addition, after a further review of the manuscript, the authors identified that the term “film” is more scientifically appropriate than “biofilm” for describing the material evaluated in this study. Therefore, the term “biofilm” has been replaced with “film” throughout the manuscript, including the title. This correction does not affect the results, discussion, or conclusions of the study and is intended solely to ensure terminological accuracy.

The authors state that the scientific conclusions are unaffected. This correction was approved by the Academic Editor. The original publication has also been updated.
